# A tale of two countries: progress towards UNAIDS 90‐90‐90 targets in Botswana and Australia

**DOI:** 10.1002/jia2.25090

**Published:** 2018-03-06

**Authors:** Tafireyi Marukutira, Mark Stoové, Shahin Lockman, Lisa A Mills, Tendani Gaolathe, Refeletswe Lebelonyane, Joseph N Jarvis, Sherrie L Kelly, David P Wilson, Stanley Luchters, Suzanne M Crowe, Margaret Hellard

**Affiliations:** ^1^ Public Health Burnet Institute Melbourne Vic. Australia; ^2^ Department of Epidemiology and Preventive Medicine Monash University Melbourne Vic. Australia; ^3^ Division of Infectious Disease Brigham and Women's Hospital Boston MA USA; ^4^ Department of Immunology and Infectious Diseases Harvard T.H. Chan School of Public Health Boston MA USA; ^5^ Division of Global Health Centers for Disease Control and Prevention Gaborone Botswana; ^6^ Botswana Harvard AIDS Institute Partnership Gaborone Botswana; ^7^ Department of HIV Prevention and Care Ministry of Health Gaborone Botswana; ^8^ Department of Clinical Research Faculty of Infectious and Tropical Diseases London School of Hygiene and Tropical Medicine London United Kingdom; ^9^ Infectious Diseases Modelling Burnet Institute Melbourne Vic. Australia; ^10^ School of Public Health and Preventive Medicine Monash University Melbourne Vic. Australia; ^11^ International Centre for Reproductive Health Ghent University Ghent Belgium

**Keywords:** HIV care continuum, 90‐90‐90 targets, HIV testing, ARV, viral suppression, Botswana, Australia

## Abstract

UNAIDS 90‐90‐90 targets and Fast‐Track commitments are presented as precursors to ending the AIDS epidemic by 2030, through effecting a 90% reduction in new HIV infections and AIDS‐related deaths from 2010 levels (HIV epidemic control). Botswana, a low to middle‐income country with the third‐highest HIV prevalence, and Australia, a low‐prevalence high‐income country with an epidemic concentrated among men who have sex with men (MSM), have made significant strides towards achieving the UNAIDS 90‐90‐90 targets. These two countries provide lessons for different epidemic settings. This paper discusses the lessons that can be drawn from Botswana and Australia with respect to their success in HIV testing, treatment, viral suppression and other HIV prevention strategies for HIV epidemic control. Botswana and Australia are on target to achieving the 90‐90‐90 targets for HIV epidemic control, made possible by comprehensive HIV testing and treatment programmes in the two countries. As of 2015, 70% of all people assumed to be living with HIV had viral suppression in Botswana and Australia. However, HIV incidence remains above one per cent in the general population in Botswana and in MSM in Australia. The two countries have demonstrated that rapid HIV testing that is accessible and targeted at key and vulnerable populations is required in order to continue identifying new HIV infections. All citizens living with HIV in both countries are eligible for antiretroviral therapy (ART) and viral load monitoring through government‐funded programmes. Notwithstanding their success in reducing HIV transmission to date, programmes in both countries must continue to be supported at current levels to maintain epidemic suppression. Scaled HIV testing, linkage to care, universal ART, monitoring patients on treatment over and above strengthened HIV prevention strategies (e.g. male circumcision and pre‐exposure prophylaxis) will all continue to require funding. The progress that Botswana and Australia have made towards meeting the 90‐90‐90 targets is commendable. However, in order to reduce HIV incidence significantly towards 2030, there is a need for sustained HIV testing, linkage to care and high treatment coverage. Botswana and Australia provide useful lessons for developing countries with generalized epidemics and high‐income countries with concentrated epidemics.

## 1 Introduction

In 2014, the Joint United Nations Programme on HIV/AIDS (UNAIDS) set the 90‐90‐90 targets to be reached by 2020 in order to end the AIDS epidemic by 2030 1, and subsequently expanded them in the Fast‐Track commitments drawn from the 2016 United Nations Political Declaration 2. The 90‐90‐90 HIV care cascade targets are that by 2020, 90% of people living with HIV (PLHIV) will be diagnosed, 90% of those diagnosed will be on antiretroviral therapy (ART), and 90% of people on treatment will achieve viral suppression. This achievement will translate to 73% of PLHIV being virally suppressed 1. Achieving 90‐90‐90 by 2020 and scaling up to 95‐95‐95 by 2030 will reduce annual new HIV infections and AIDS‐related deaths by 90% compared to 2010 3.

The 90‐90‐90 targets focus solely on HIV testing, ART scale‐up and viral suppression while the Fast‐Track approach includes rapid scale‐up of effective prevention and treatment services as well as zero discrimination 2. Prevention strategies captured in the Fast‐Track approach include eliminating new infections among children, improving access to combination HIV prevention options including condom programming, pre‐exposure prophylaxis (PrEP), and voluntary medical male circumcision (VMMC) in priority countries; eliminating gender inequalities and violence; focusing on young people, and HIV‐sensitive social protection; shifting towards community‐based service delivery; investing in HIV prevention and social enablers; addressing human rights issues for PLHIV and those at risk; and improving universal health coverage, including treatment for tuberculosis, cervical cancer and hepatitis B and C 2.

Significant global scale‐up of HIV testing, treatment and viral load monitoring is required to achieve the 90‐90‐90 targets by 2020. In 2015, of 36.7 million PLHIV, only 57% were diagnosed, 46% on ART, and 38% virally suppressed. With further progress in 2016, 70% were diagnosed, 53% on treatment and 44% virally suppressed 4, 5. Viral suppression is thus significantly below the 73% target, hence a concerted effort is still required from individual countries in order to meet the global target.

Many countries are making significant progress towards 90‐90‐90 targets 4, but progress varies due to the heterogeneity of local epidemics and health system capacity. Unfortunately, countries with high disease burden often have limited resources and infrastructure. Sweden, a high‐income country with a centralized healthcare system and governance and low HIV burden was the first country to achieve the 90‐90‐90 targets in 2016 6. However, several low‐income high‐HIV‐burden countries are now close to achieving the targets: Swaziland, a low‐income, high‐burden country, reported 68% viral suppression among PLHIV in 2016 5.

Botswana and Australia, with markedly different epidemics, income levels, and HIV budgets, are close to achieving the 90‐90‐90 targets with robust HIV treatment programmes for a long time now. Botswana has a generalized HIV epidemic while Australia has a concentrated epidemic in men who have sex with men (MSM). Should the two countries achieve the desired 90% reduction in new HIV infections and end AIDS‐related deaths by 2030, valuable lessons can be learned. This commentary compares the HIV epidemics in these countries, their progress towards achieving the 90‐90‐90 targets alongside HIV incidence, and identifies lessons for HIV epidemic control in similar settings.

## 2 Discussion

Botswana and Australia are on target to achieve the 90‐90‐90 targets for HIV epidemic control due to successful HIV testing and treatment programmes. Their progress towards the 90‐90‐90 targets and status of epidemic control is discussed below. Lessons learned from HIV testing, ART coverage, sustained viral suppression, financing the HIV response and monitoring the 90‐90‐90 targets are explored. Table 1 summarizes key achievements and gaps of HIV care delivery in both settings.

**Table 1:** Summary of key HIV care delivery achievements and gaps

	Australia		Botswana	
	Achievements	Gaps	Achievements	Gaps
HIV testing	High HIV testing coverage Free or low‐cost HIV testing Point‐of‐care HIV testing and laboratory based confirmatory testing available Increasing HIV testing frequency in risk populations	Up‐front cost incurred for some migrant groups i.e. those ineligible for government funding Suboptimal rates of high‐frequency HIV testing among high risk groups No federal government subsidy and limited coverage of rapid testing service models Lack of home testing options	High HIV testing coverage Free HIV testing Point‐of‐care diagnostic HIV testing available Community‐based HIV testing available	Pockets of low rates of HIV testing among men and young people Few HIV testing/treatment services tailored for key populations (e.g. MSM, FSW)
HIV treatment & monitoring	Government‐funded HIV treatment programmes Adopted universal treatment for all PLHIV High ART coverage Routine viral load monitoring available High viral suppression among people on ART Declining HIV‐related mortality	Up‐front cost incurred for some migrant groups	Free ART for citizens, government‐funded HIV treatment programmes Adopted universal treatment for all PLHIV High ART coverage Routine viral load monitoring available High viral suppression among people on ART Declining HIV‐related mortality Decentralized healthcare system with lowest facility levels offering HIV care	Free ART not available to immigrants Lack of optimal integration of HIV treatment services e.g. with maternal child health, tuberculosis, sexually transmitted infections HIV treatment is only facility based and no widespread community‐based HIV treatment and monitoring programmes
Programmatic and prevention responses	Pilot PrEP programmes available even though not accessible to some migrants	No clear downward trend of HIV incidence in MSM	PrEP implementation available but limited to a few sites	HIV incidence remained above 1% in 2015 Low uptake of male circumcision

### 2.1 90‐90‐90 target status in Botswana and Australia

The status of the 90‐90‐90 targets in Botswana is drawn from a population‐based survey in rural and peri‐urban Botswana (2013 to 2015): 83.3% (81.4% to 85.2%) of PLHIV were diagnosed, with 87.4% (85.8% to 89.1%) on ART and 96.5% (96% to 97%) of those virally suppressed 7. Overall, 70.2% of PLHIV were virally suppressed (Table 2). It is important to note that these are recent estimates from an intensive effort to enrol a representative, population‐based sample; however, some residents did not enrol (usually due to being absent from home), and the study did not include urban populations. Sensitivity analyses in this study 7 to assess the potential of non‐participation for the observed 90‐90‐90 estimates are highlighted in Table 2 (showing lower but still relatively high 90‐90‐90 coverage estimates). In 2015, modelling using Australian surveillance data and direct observations produced estimates that 90% of PLHIV were diagnosed, with 84% on ART and 92% of those virally suppressed 8. Overall, 69.5% of PLHIV in Australia were virally suppressed (Table 2). The main difference in HIV treatment cascades is higher HIV testing coverage in Australia and higher ART coverage in Botswana, with overall viral suppression of around 70% in both countries, just below the 73% target. However, according to the 2017 UNAIDS report, Botswana has now exceeded the viral suppression target, reaching 78%, while Australia is nearing the target and is at 71% 5. Such high levels of viral suppression should translate into reduction in HIV incidence over time and ultimately epidemic control.

**Table 2:** Demographics and status of UNAIDS 90‐90‐90 targets

Profile	Australia	Botswana
Demographic profile		
Population	24,051,400 9	2,024,904 10
Non‐citizens/immigrants	6,900,000 9	350,000 10
Gross domestic product (GDP)	1.339 trillion USD 11	14.39 billion USD 12
Disease profile (HIV)		
Type of HIV epidemic	Concentrated	Generalized
Estimated PLHIV	25,313 8	392,432 13
HIV prevalence	0.1% 8	18.5% 14
Annual HIV incidence	0.04% 8	1.34% 15
First “90” (proportion of est. PLHIV knowing their HIV status)	90% 8	83.3% (81.4% to 85.2%) 7 Adjustment for non‐participation by unenrolled, eligible household members: 82.8% (80.9% to 84.7%). Standardization to Botswana nationwide census age and gender distribution: 77.8% (76.2 to 79.4)
Second “90” (proportion of diagnosed on ART)	84% 8	87.4% (85.8 to 89.0) 7 Adjustment for non‐participation by unenrolled, eligible household members: 87.4% (85.8 to 89.1). Standardization to Botswana nationwide census age and gender distribution: 85.0% (83.3 to 86.8)
Third “90” (proportion of those on ART with viral suppression)	92% 8	96.5% (96.0 to 97.0) 7 Adjustment for non‐participation by unenrolled, eligible household members: 96.5% (96.0 to 97.0). Standardization to Botswana nationwide census age and gender distribution: 93.9% (92.4 to 95.3)
Overall viral suppression in PLHIV^a^ (proportion of est. PLHIV with viral suppression)	69.5% 8	70.2% (67.5 to 73.0) 7 Adjustment for non‐participation by unenrolled, eligible household members: 69.8% (67.1 to 72.6). Standardization to Botswana nationwide census age and gender distribution: 63.4% (61.6 to 65.1)

a A recent UNAIDS report categorizes Botswana as having achieved the 90‐90‐90 targets with 78% overall viral suppression and Australia as close to the 73% target (68% to 72.5%) 5.

### 2.2 HIV epidemic control in Botswana and Australia

Trends in HIV incidence and mortality can be used to track progress towards HIV epidemic control. Botswana and Australia have reported substantial reductions in HIV‐related mortality. HIV‐related mortality in Australia dropped from approximately 500 deaths in 2010 to 200 in 2015, and for Botswana from 5300 in 2010 to 3200 16. While reaching or exceeding the 90‐90‐90 targets should ensure mortality rates decrease further, in Botswana in particular, co‐infections with tuberculosis, other opportunistic infections and HIV‐associated cancers 17 need to be addressed if progress is to be maintained.

In Botswana, there is no evidence that general population HIV incidence estimates have changed substantially since 2008 [1.45% (95% confidence interval (CI) 0.66 to 2.24) in 2008 and 1.34% (95% CI 0.91 to 1.77) in 2015] 14, 15, 18, although 95% confidence intervals around these estimates are large. In Australia's concentrated epidemic, estimates of annual HIV incidence among MSM peaked in 2011 at 1.32% (1.03 to 1.70), declined to 0.65% (0.50 to 0.86) by 2013, before rising to 0.89% (0.69 to 1.14) by 2015 8.

According to UNAIDS, achieving 90‐90‐90 targets by 2020 (in conjunction with scale‐up of HIV prevention modalities) will be followed by HIV epidemic control in the subsequent 10 years. It is thus likely too early to assess the impact of approaching 90‐90‐90 targets on incidence trends in Botswana (assuming that such high levels of testing and treatment coverage were only recently realized). In addition, other potential reasons for persistence of high HIV incidence in Botswana include a substantial proportion of PLHIV without viral suppression (~30% of PLHIV) in a population with very high‐HIV prevalence, and pockets of lower testing, treatment coverage and viral suppression in high‐risk groups such as men and young people 7, 19. In Australia, HIV incidence is probably driven by a combination of HIV transmission from MSM with undiagnosed HIV and delayed diagnosis in other sub‐populations 20.

Modelling suggests the greatest reductions in HIV incidence from improvements in the HIV treatment cascade will occur in regions with the greatest gaps in HIV testing and ART coverage 21. Despite high existing testing and ART coverage in Botswana and Australia, efforts to address the commitments/targets required to end AIDS by 2030 highlighted in the Fast‐Track targets, including those related to primary prevention, should be intensified 2. Modelling by Scott et al. (2017) shows Australia will need to surpass the 90‐90‐90 targets, increase HIV testing frequency, and increase MSM PrEP coverage and condom use to 100% in order to achieve 80% HIV incidence reduction by 2030. The model suggests that many countries – especially those with low HIV prevalence – will struggle to reduce HIV incidence by 90% by 2030 even if the UNAIDS 90‐90‐90 targets are met 22. In such cases, HIV prevention should be the focus and this includes scaling up HIV testing, including increased frequency of testing in key and vulnerable populations, and promoting prevention approaches such as PrEP.

### 2.3 Lessons learned

#### 2.3.1 HIV testing

HIV testing remains the most important step in HIV prevention and care. HIV diagnostic approaches in the two countries differ. Botswana utilizes point‐of‐care HIV testing (POCT) with two rapid HIV tests at clinics, hospitals, community‐based organizations, testing centres and mobile testing campaigns. In Australia, POCT with rapid tests is used for screening; laboratory‐based diagnostic testing is required. HIV testing in Australia occurs through public sexual health and general practitioners’ clinics and hospitals.

Rapid testing uptake in Australia has been modest, with lack of medical insurance reimbursements for tests deterring use of POCT. Strategies to enhance the reach and frequency of HIV testing must recognize local needs, with consideration of HIV distribution by risk populations, geography and system capacity. Australia's concentrated epidemic, high coverage of primary care services in urban areas and public subsidy of testing has meant 90% of PLHIV know their status 8. In Botswana, POCT delivered across clinical, programme and community settings suits a more generalized and geographically dispersed epidemic.

Botswana and Australia have demonstrated that POCT needs to be accessible and targeted at key and vulnerable populations to identify new HIV infections. In Australia, MSM, people who inject drugs (PWID) and sex workers are key populations, while indigenous populations are considered vulnerable 8. In Botswana, female sex workers (FSWs), MSM and non–citizens (illegal immigrants) are key populations, while adolescents/young people are considered vulnerable and the testing rate is low in men 16, 18. Botswana has expanded HIV testing beyond facility‐based testing to involve communities using community‐based HIV testing campaigns.

To progress, within countries, systems need to respond to changes in epidemiology and political and structural environments. Identification of key and vulnerable populations and then enhancing systems that increase HIV testing, linkage to care and treatment in these groups is key. In Australia, recent outbreaks of HIV among indigenous people and PWID 8 suggest that new strategies are needed to reach these groups. Similarly, in Botswana the ongoing targeting of FSWs and increasing recognition of MSM as a key population may require adapted models of testing to maintain and increase diagnosis among PLHIV, such as the integration of HIV self‐testing into existing programmes.

#### 2.3.2 ART coverage

Botswana adopted the World Health Organization's (WHO) “treatment for all” strategy (universal ART) 22 in mid‐2016 23, whereas Australia formally implemented universal ART policy in 2014 24. Interestingly, Botswana had a similarly high estimated ART coverage (87%, vs. 84% in Australia) among persons with diagnosed HIV infection even before offering universal ART 7, 8.

Botswana offers free ART to citizens through all levels of its decentralized healthcare system and excludes immigrants. In Australia, ART is provided through GPs’ clinics, sexual health clinics, and hospitals for citizens and permanent residents, publicly funded through Australia's universal healthcare system with low direct costs for patients. Immigrants living with HIV holding short‐term visas are not covered, but generally access ART through compassionate programmes.

Universal ART access in Botswana and Australia – combined with initiation of treatment in community settings – promotes rapid ART initiation and reduced periods of infectivity. Both healthcare systems must prepare for challenges associated with universal ART, including drug resistance, adherence and retention, particularly in marginalized subgroups such as immigrants, refugees and PWID. In Botswana, a decentralized non‐siloed healthcare system improves ART coverage; integration of ART into maternal, newborn and child health services, and tuberculosis treatment to reduce multiple visits to services, could further enhance effectiveness 25.

#### 2.3.3 Achieving sustained viral suppression

Viral suppression rates for people on ART (defined as <400 HIV RNA copies/ml in Botswana 23 and <200 HIV RNA copies/ml in Australia 24) are very high and above 90% in both countries (Table 2). Six‐monthly viral load monitoring is recommended and available for stable patients on treatment in both countries 23, 24. As WHO guidelines specify, Australia and Botswana have routine access to viral load monitoring in their public health system, unlike many resource‐limited settings 26.

Sustained viral suppression is driven by adherence to ART and absence of drug resistance. Enablers and disablers of adherence should be identified and addressed. In Australia, social/economic/cultural/patient engagement factors are associated with suboptimal adherence while ART regimen/clinical factors are not 27. Community‐based programmes that enhance the continuum of care could also benefit adherence and sustained viral suppression 28, assisting both countries in achieving the 2030 targets.

#### 2.3.4 Financing the HIV response

Governments and/or donors must provide sustainable HIV response financing. Scaled HIV testing, linkage to care, universal ART, monitoring patients on treatment, stronger condom programming and biomedical prevention tools (e.g. VMMC, PrEP) all require funding with strong human resources and supply chain management.

Australia has a GDP of $1.3 trillion, while Botswana's is $14.3 billion (Table 2). This difference in GDP impacts on the funding pathways for the HIV response: in Australia, the HIV response is predominantly funded by government 24; in Botswana, the government funds at least 60% of its HIV response budget, with the balance from international and private funding 19. A decline in international funding for Botswana, because of its new upper middle‐income status 29 and an overall adverse global economic environment for aid, highlights the vulnerability of its HIV response 19. Cost‐benefit assessments may identify optimal approaches to the HIV response, but the cost relative to a country's budget is often critical. Innovative funding strategies, combined with improved efficiency, will be required to ensure Botswana and similar countries can sustain high‐level HIV responses 30.

#### 2.3.5 Monitoring the 90‐90‐90 targets

Reliable data sources and standardized methodologies and definitions are required to accurately report progress towards the 90‐90‐90 targets 31, however, considerable disparities in reporting methods exist. Use of complete population‐based datasets from clinical elements of a centralized healthcare system to report the HIV testing and treatment cascade has been proposed 32.

Botswana's reported progress towards targets is based on an ongoing population survey of adult members of 20% of households in 30 rural and peri‐urban communities (nested within a clinical trial of interventions targeted at enhancing HIV testing, linkage and treatment), with sensitivity analyses including standardization to age/sex distribution from census and HIV surveillance data 7. Despite intensive efforts to achieve high survey participation, these data may not be representative of the entire population, and non‐participants may differ from survey participants in key ways. In Australia, state‐based and centralized national programmes monitor and report HIV data 8. Population‐level data from surveillance embedded within inter‐linked rather than stand‐alone health services would be ideal, but such approaches are costly and sometimes impractical.

## 3 Conclusions

While Botswana's and Australia's progress towards the UNAIDS 90‐90‐90 targets is commendable, HIV incidence remains high. The hope for these two countries and others is that sustained testing and treatment coverage will contribute substantially toward epidemic control in the coming years. The Fast‐Track approach encourages countries to rethink and re‐emphasize primary HIV prevention, implement other strategies such as PrEP, scale up HIV testing (including HIV self‐testing) and link PLHIV to care and treatment. HIV prevention efforts need to be reinvigorated if epidemic control is to be achieved.

## Competing interests

TM, MS, SLK, LAM, TG, RL, JNJ, SLK, DPW, SL, SMC, MH: No competing interests to declare.

## Authors’ contributions

TM, MS and MH developed the concept, drafted, reviewed and approved the manuscript. SL, LAM, TG, RL, JNJ, SK, DW, SL and SMC contributed to the manuscript review and approval.

## Funding

There was no direct funding for this work; resources were provided through Burnet Institute institutional funds for TM.

TM is a recipient of an Australian Government PhD scholarship. MH, MS and SMC are the recipients of National Health and Medical Research Council fellowships. The Burnet Institute receives support from the Victorian Government Operational Infrastructure Support Programme.

## Disclaimer

The findings in this paper are those of the authors and do not necessarily represent the official position of the funding agencies.
